# Chemical composition and in vitro fermentation characteristics of legumes using canine fecal inoculum

**DOI:** 10.1093/tas/txaa200

**Published:** 2020-10-30

**Authors:** Zachary T Traughber, Fei He, Jolene M Hoke, Gary M Davenport, Maria R C de Godoy

**Affiliations:** 1 Department of Animal Sciences, University of Illinois, Urbana, IL; 2 Archer Daniels Midland Company, Decatur, IL

**Keywords:** dog, dietary fiber, in vitro fermentation, legumes, nutrient composition

## Abstract

Legumes are a popular grain-free alternative carbohydrate source in canine diets, however, information on their fermentative characteristics have not been established. Thus, the objectives of the present study were to 1) quantify the chemical compositions and 2) fermentative profile of select legumes using canine fecal inoculum. Five legume varieties, whole yellow peas (**WYP**), green lentils (**GL**), black bean grits (**BBG**), navy bean powder (**NBP**), and garbanzo beans, were analyzed and compared to a positive control, beet pulp (**BP**). Substrates were analyzed for gross energy (**GE**), dry and organic matter, crude protein (**CP**), acid hydrolyzed fat, and total dietary fiber (**TDF**) fractions, beta-glucans, starch-free, and hydrolyzed sugars, as well as fermentative characteristics: pH, short-chain fatty acids (**SCFA**), branched-chain fatty acids (**BCFA**), total gas, hydrogen, and methane. Substrates then underwent a two-stage in vitro digestion and subsequent fermentation using canine fecal inoculum for 0, 3, 6, 9, and 12 h. All test substrates contained approximately 8% to 9% moisture and 4.5 kcal/g GE. The highest CP content was observed in GL (27%). Analyzed TDF content of test substrates was greatest for WYP (32%) and GL (36%). Total starch content was greatest for GL (58%) and WYP (56%). Sucrose and stachyose were the most predominant free sugars and glucose was the most predominant hydrolyzed sugar among test substrates. After 3 and 6 h of fermentation, a net negative change in pH was observed among most substrates with a net negative change in all substrates after 9 and 12 h. Values for SCFA did not differ among substrates after 3 or 6 h of fermentation with BP and WYP among the greatest acetate (1,656 and 1,765 umol/g, respectively) and propionate production values (157.7 and 126.1, respectively) after 9 h. All substrates produced greater total gas volumes than WYP after 3 h, with no differences observed after any other time points. However, BP hydrogen production values were greater after 9 and 12 h (*P* < 0.0001; 726,042 and 394,675 ng/g, respectively) with greater methane production values after 12 h (*P* < 0.0001; 54,291 ng/g) than all test substrates. These data suggest that legumes offer a diverse macronutrient profile and appear to be a source of slowly fermentable fiber, which may have beneficial implications on the ratios of saccharolytic to proteolytic fermentation toward the distal colon.

## INTRODUCTION

Recent controversies with grain-free canine diets have sparked interest in the safety and efficacy of popular plant-based protein and carbohydrate sources. Legumes are rich in galactosyl-sucrose oligosaccharides such as raffinose, stachyose, and verbascose (Saini, 1989). The digestibility of various legumes in a canine model has been evaluated (Carciofi et al., 2008; Forster et al., 2012; Reilly et al., 2019), however, information on fermentative characteristics of some legume varieties are lacking (Bednar et al., 2001).

Bacteria ferment carbohydrates and nitrogen compounds that pass undigested into the large intestine producing metabolites that can be utilized by the host (Cummings and Macfarlane, 1991). The end products of microbial fermentation may lower intestinal pH and thus, prevent the overgrowth of pH-sensitive pathogenic bacteria (Topping and Clifton, 2001). Proteolytic fermentation, however, leads to the production of detrimental metabolites such as branched-chain fatty acids (**BCFA**), phenolic and indolic compounds, sulfides, and ammonia that have been associated with colon and colorectal cancers (Bingham, 1988; Hughes et al., 2000, Carbonero et al., 2012), exacerbation of ulcerative colitis (Gibson et al., 1991; Ramakrishna et al., 1991), and inflammatory bowel disease in humans (Carbonero et al., 2012), skin cancer in mice (Boutwell and Bosch, 1959), and the promotion of tumorigenesis in rats (Visek, 1978; Clinton et al., 1988).

Three-stage in vitro models have been used widely to assess fermentative characteristics of ingredient sources and diets using either ileal digesta (Murray et al., 2001; Bosch et al., 2008) or freshly voided feces (Flickinger et al., 2000; Swanson et al., 2001; Vickers et al., 2001; Faber et al., 2011). Most fermentation occurs in the proximal, ascending portion of the large intestine, where substrate availability is highest (Cummings et al., 1987; Macfarlane et al., 1992). Actual location, proximal or distal colon, of substrate fermentation by microbes is dependent on multiple factors such as intestinal transit time and the speed at which each substrate is fermented, and thus, the in vitro fermentation kinetics offers valuable insights into the potential effects of substrates in an in vivo model.

The objectives of the current study are 1) to establish macronutrient and carbohydrate profiles and 2) to quantify and compare the fermentation characteristics of select legumes that may provide beneficial effects to host health.

## MATERIALS AND METHODS

The protocol used in this study was approved by the Institutional Animal Care and Use Committee at the University of Illinois at Urbana-Champaign. All methods were performed in accordance with the U.S. Public Health Service Policy on Humane Care and Use of Laboratory Animals.

### Ingredient Samples

Five legume varieties were analyzed: whole yellow peas (**WYP**), green lentils (**GL**), black bean grits (**BBG**), navy bean powder (**NBP**), and garbanzo beans (**GB**; Archer Daniels Midland Company, Decatur, IL) and compared to a positive control, beet pulp (**BP**; Tri State Packaging Co, Van Wert, OH).

### Sample Preparation and Chemical Analysis

Ingredient samples were ground in a Wiley mill (model 4; Thomas Scientific, Swedesboro, NJ) through a 1-mm screen with dry ice to prevent sample heating. Diet and fecal samples were analyzed for dry matter (**DM**), organic matter (**OM**), and ash according to AOAC (2006; methods 934.01 and 942.05). Crude protein (**CP**) content of the diets and fecal samples was calculated from Leco (TruMac N, Leco Corporation, St. Joseph, MI) total nitrogen values according to AOAC (2006; method 992.15). Acid hydrolyzed fat (**AHF**) was determined by acid hydrolysis followed by ether extraction according to the methods of the American Association of Cereal Chemists (1983) and Budde (1952). Total (**TDF**) and insoluble dietary fiber (**IDF**) contents were analyzed according to Prosky et al. (1992) and soluble fiber (**SDF**) was determined by the difference. Ingredient samples were assayed for gross energy (**GE**) by bomb calorimeter (Model 6200, Parr Instruments Co., Moline, IL). Substrates were analyzed in duplicate and values were reported on a dry matter basis (**DMB**).

Free sugar profiles of substrates were determined according to methods of Churms (1982), and Kakehi and Honda (1989) by the Experiment Station Chemical Laboratories (Columbia, MO). Hydrolyzed sugar profiles were determined according to the methods of Oxley et al. (2004), Hudson et al. (1982), Kiho et al. (1986), and Fox et al. (1989) by the Experiment Station Chemical Laboratories (Columbia, MO). Free sugar profiles of substrates following first- and second-stage digestion were analyzed by ion chromatography with pulsed amperometric detection (IC-PAD) on a Thermo Scientific Dionex ICS-5000 system (Sunnyvale, CA), equipped with a quaternary gradient pump, a thermo AS-AP autosampler, and an electrochemical detector with a cell containing an Ag/AgCl reference electrode and a disposable gold/PTFE working electrode. Analytes were separated on a Thermo Scientific CarboPac PA1 column (4 mm ID × 250 mm; Dionex, USA) in combination with a CarboPac PA guard column (4 mm ID × 50 mm) operated at 30 °C and values from each set of duplicates were averaged.

Resistant, non-resistant, and total starch were analyzed using a resistant starch kit (Megazyme, Wicklow, Ireland) according to AOAC (2006; method 2002.02). Cereal β-glucans were analyzed using the McCleary method (McCleary and Codd, 1991; McCleary and Mugford, 1997) using a β-glucan kit (Megazyme, Wicklow, Ireland) according to AOAC (2000; method 995.16).

### In Vitro Fermentation Procedures

**Figure 1.**
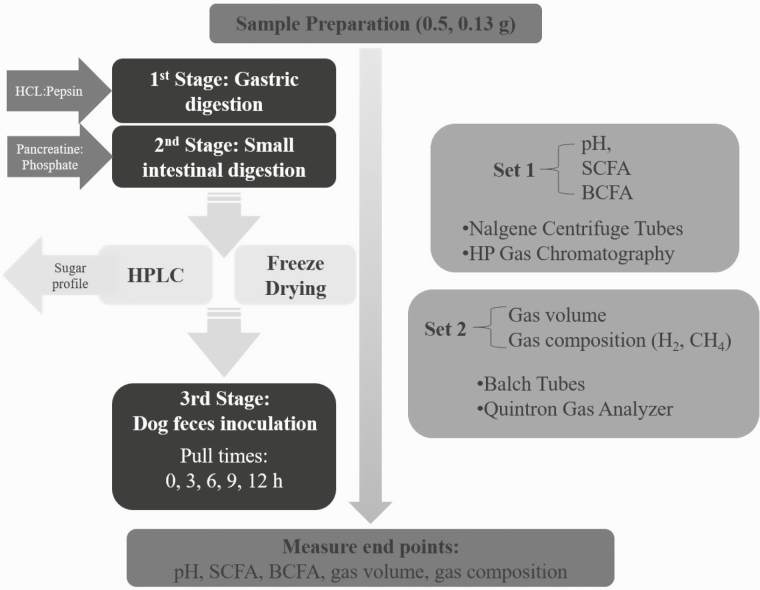
In vitro fermentation procedure based on methods of Bourquin et al. (1993). Two sets of fermentations were conducted in triplicate for each sample. Sample aliquots (0.5 g for Set 1 and 0.13 g for Set 2) were first digested for 6 h with a phosphate-buffered HCL and pepsin solution (pH ~2), followed by a phosphate buffered pancreatin for 18 h. Free sugar profiles were analyzed and representative sugar blanks were developed and digested samples were lyophilized. Fecal bacteria for inoculation were composited from freshly voided samples of three female beagles and added to medium as described by Bourquin et al. (1993). Freeze-dried samples were inoculated with canine fecal bacteria, flushed with CO_2_, and incubated at 39°C for 0, 3, 6, 9, or 12 h and immediately submerged in an ice bath to abruptly stop the fermentation process. Samples then were analyzed for pH, and subsequently analyzed for SCFA and BCFA (Set 1), and gas volume and composition (Set 2).

Two sets of fermentations were conducted in triplicate for each sample in completely anaerobic environments via flushing with oxygen-free carbon dioxide; the first set measured pH and volatile fatty acids in 50 mL polypropylene centrifuge tubes fitted with one-way pressure-release stoppers, and the second set measured gas volume and composition using 25 mL Balch tubes, stoppered and crimp-sealed to prevent the release of gasses (Bourquin et al., 1993). Ingredient sample aliquots (0.4995 to 0.5000 g for Set 1 and 0.1295 to 0.1300 g for Set 2) were first digested in vitro with pepsin (MiliporeSigma, St. Louis, MO) buffered in HCL–phosphate solution (pH ~2) for 6 h, followed by a second digestion with pancreatin (MilliporeSigma, St. Louis, MO) buffered in phosphate solution for 18 h to mimic gastric and small intestinal digestion, respectively (Figure 1). Chloramphenicol (MilliporeSigma, St. Louis, MO) was added to one of two groups of tubes from both sets of tubes for each substrate to verify the absence of microbial activity within substrates prior to third stage digestion/fermentation. Liquid portions of these sets were filtered through Amicon Ultra-3 10K centrifugal filter devices (MilliporeSigma, St. Louis, MO) for protein removal prior to free sugar profile analysis. Free sugar profiles of filtered substrates were quantified, and similar profiles were developed using inositol, sorbitol, galactose, glucose, xylose, sucrose, and fructose for each substrate and were used as respective representative sugar blanks during the third stage fermentation. The remaining unfiltered, digested samples, then, were lyophilized in an FTS Systems Dura-Dry MP freeze-dryer (SP Scientific, Warminster, PA).

Fecal bacteria were obtained and composited from equal aliquots of freshly voided samples from two female beagles consuming a high-protein, high-fiber extruded diet, added to and blended with medium as described by Bourquin et al. (1993), and passed through a quadruple-layer of sterile cheesecloth. Freeze-dried samples were inoculated with canine fecal bacteria, flushed with CO_2_, and incubated at 37°C for 0, 3, 6, 9, or 12 h and immediately submerged in an ice bath to abruptly stop the fermentation process. Samples, then, were analyzed for pH, and subsequently analyzed for short-chain fatty acids (**SCFA**), BCFA, and gas volume and composition. For each time point, one set of tubes devoid of substrate was inoculated and fermented to allow for correction of residual SCFA, BCFA, and gas production unrelated to substrate fermentation. Subsequent acetate, propionate, butyrate, isobutyrate, isovalerate, and valerate production values from each set of triplicate tubes were averaged.

Concentrations of SCFA and BCFA in the diluted samples were determined according to the methods of Erwin et al. (1961) and Goodall and Byers (1978) using a Hewlett-Packard (HewlettPackard, Avondale, PA) Model 5890A gas chromatograph equipped with a flame ionization detector (FID) on a column (1.8 m × 4 mm ID) packed with GP 10% SP-1200/1% H_3_PO_4_ on 80/100 chromosorb W AW (Supelco, Bellefonte, PA). The carrier gas was nitrogen, with a flow rate of 45 mL/min. The oven, injection port, and detector port temperatures were 125, 175, and 180 °C, respectively.

### Statistical Analysis

Triplicate values were analyzed using PROC MIXED in SAS (SAS Institute Inc., version 9.4, Cary, NC). Normality of residuals was analyzed using PROC UNIVARIATE. Abnormal data (propionate, butyrate, and isovalerate) underwent a logarithmic transformation to achieve normality using the following equation: *Y* = Log_10_ (*X* + 1), whereby “*Y*” represents the transformed value and “*X*” represents the raw value. Statistical significance was evaluated using normally distributed data (raw or transformed as necessary). Differences among substrates, time, and substrate*time were determined using a Fisher-protected least significant difference test with a Tukey adjustment to control for type-1 experiment-wise error. Data were presented as least squares means. Statistical significance was set at *P* < 0.05 and trends defined as 0.05 < *P* < 0.10.

## RESULTS

### Nutrient Composition of Test Substrates

All test substrates contained approximately 8% to 9% moisture and 4.5 kcal/g **GE** (Table 1). Ash values were similar among substrates with BBG containing the highest concentration of 3.8%. The highest CP content was observed in GL (27.1%), and the lowest was observed in NBP (20.8%). All substrates except for GB contained similar AHF contents of approximately 3% with GB containing the highest concentration (6.9%). Analyzed TDF contents of WYP and GL (31.8% and 35.8%, respectively) were greater than values for BBG, NBP, and GB, each of which contained approximately 25%. The highest IDF concentrations were observed in WYP and GL (33.2% and 31.6%, respectively) and the lowest concentrations were observed in BBG and NBP (19.4% and 16.9%, respectively). Both BBG and NBP also contained the highest concentrations of SDF among test substrates (5.8% and 7.5%, respectively) and GL contained virtually none (0.1%).

**Table 1.** Chemical composition of select legumes

	Treatment				
Ingredient, %	WYP	GL	BBG	NBP	GB
Moisture	9.1	10.3	8.3	8.0	8.3
	*%, Dry matter basis*				
GE, kcal/g	4.5	4.5	4.5	4.4	4.7
Ash	2.7	2.6	3.8	3.2	3.2
CP	23.0	27.1	25.9	20.8	22.8
AHF	2.4	2.5	3.1	3.9	6.9
IDF	33.2	31.6	19.4	16.9	23.7
SDF^1^	1.7	0.1	5.8	7.5	2.2
TDF	34.8	31.8	25.2	24.4	25.5

^1^SDF = TDF – IDF.

No test substrates had detectable levels (>0.35%) of cereal beta-glucans (Table 2). Both GL and WYP had the highest total starch contents (57.5% and 56.4%, respectively), BBG had the lowest (39.6%), and NBP and GB were intermediate (45.0% and 49.7%, respectively) among test substrates (Table 2). A similar pattern also was observed when non-resistant starch was quantified. The greatest resistant starch concentrations were observed in WYP and NBP with approximately 4% each.

**Table 2.** Beta-glucan and starch concentrations of select legumes

Ingredient^1^	WYP	GL	BBG	NBP	GB
Beta-glucan	n.d.^3^	n.d.	n.d.	n.d.	n.d.
*Starch*					
Resistant	4.02	2.58	3.52	3.89	1.02
Non-resistant	52.35	54.92	36.09	41.13	48.68
Total^2^	56.37	57.50	39.61	45.02	49.70

^1^W/W% = grams per 100 g of sample.

^2^Total starch = resistant + non-resistant starch.

^3^Non-detectable (<0.35).

Sucrose and stachyose were the two predominant analyzed free sugars in the test substrates (Table 3). The highest concentration of sucrose was observed in GB (4.3%) with the lowest in GL (1.5%). The highest concentrations of stachyose were observed in BBG and NBP (3.0% and 2.9%, respectively) and the lowest concentration was observed in GB (1.7%). The third-most dominant free sugar observed was verbascose in WYP and GL (1.2% and 1.1%, respectively), maltose in BBG (0.6%), raffinose in NBP (0.4%), and fructose in GB (1.4%). Glucose was the most predominant sugar in all test substrates following hydrolysis with WYP, GL, and BBG containing the highest concentrations in test substrates (5.1%, 5.9%, and 5.7%, respectively; Table 3). Mannose was the second-most prevalent hydrolyzed sugar in WYP, GL, and GB (1.5%, 0.9%, and 1.2%, respectively); however, the second-most dominant hydrolyzed sugars for BBG and NBP were galactose and arabinose (0.9% each). No detectable amounts of rhamnose or fucose were observed in any substrates, with trace amounts of ribose and xylose observed in only a few test substrates.

**Table 3.** Free and hydrolyzed sugar concentrations of select legumes

Ingredient^1^	Treatment				
	WYP	GL	BBG	NBP	GB
Moisture	9.12	10.30	8.34	7.99	8.30
*Free sugars*					
Fructose	0.21	0.97	0.07	0.07	1.40
Glucose	0.76	0.81	0.08	0.07	0.25
Sucrose	3.54	1.52	2.81	2.51	4.32
Lactose	0.06	0.04	0.00	0.00	0.10
Maltose	0.65	0.75	0.55	0.00	0.11
Raffinose	0.15	0.23	0.44	0.41	0.74
Stachyose	2.54	2.44	2.96	2.89	1.72
Verbascose	1.21	1.07	0.13	0.11	0.10
*Hydrolyzed sugars*					
Rhamnose	0.00	0.00	0.00	0.00	0.00
Ribose	0.15	0.10	0.13	0.00	0.00
Fucose	0.00	0.00	0.00	0.00	0.00
Arabinose	0.51	0.45	0.62	0.88	0.51
Xylose	0.15	0.00	0.11	0.00	0.00
Mannose	1.53	0.90	0.88	0.00	1.24
Glucose	5.13	5.94	5.71	1.68	3.46
Galactose	1.18	0.84	0.94	0.58	0.91

^1^W/W% = grams per 100 g of sample, dry matter basis.

### Metabolite Production via Third-Stage In Vitro Fermentation

#### pH change.

A significant treatment by time interaction (*P* < 0.0001) was observed for pH changes (Table 4). A net negative change in pH was observed in GL and NBP after 3 h of fermentation (–0.09 and –0.07, respectively) and for WYP, GL, BBG, and NBP after 6 h of fermentation (–0.20, –0.08, –0.07, and –0.19, respectively). A net negative change in pH was observed in all test substrates after 9 and 12 h of fermentation. pH change values of WYP, GL, and GB were similar (*P* > 0.05) to BP after all time points. After 12 h of fermentation, the reduction in pH of BBG and NBP (–0.87 and –0.96, respectively) was greater (*P* = 0.0002 and *P* < 0.0001, respectively) than the positive control, BP (–0.42).

**Table 4.** In vitro pH change and volatile fatty acid production^1^ of select legumes after 3, 6, 9, and 12 h using canine fecal inoculum

	Treatments							*P*
	BP	WYP	GL	BBG	NBP	GB	SEM^2^	Trt × time
pH change								
3 h	0.13^b^	0.25^a^	–0.09^a,b^	0.06^b^	–0.07^a,b^	0.09^b^	0.056	<0.0001
6 h	0.00^a,b^	–0.20^b^	–0.08^a,b^	–0.07^a,b^	–0.19^a,b^	0.11^a^		
9 h	–0.33^a^	–0.81^b^	–0.52^a,b^	0.30^a^	–0.30^a^	–0.38^a^		
12 h	–0.42^a,b^	–0.69^b,c^	–0.62^a,b^	–0.87^c^	–0.96^c^	–0.38^a^		
*Short-chain fatty acid*								
Acetate								
3 h	89.0	212.1	109.1	226.9	135.1	0.0	105.97	<0.0001
6 h	260.1	313.1	406.9	163.7	57.6	208.5		
9 h	1,655.6^a^	1,764.6^a^	1,319.0^a,b^	921.3^b^	961.0^b^	1,259.1^a,b^		
12 h	2,953.0^a^	2,425.7^a,b^	2,770.2^a,b^	2,895.0^a,b^	2,207.1^b^	1,571.7^c^		
Propionate								
3 h	0.0	0.0	0.0	0.0	53.2	0.0	242.75	<0.0001
6 h	0.0	0.0	42.5	0.0	0.0	13.9		
9 h	157.7^a^	126.1^a,b^	32.2^a,b,c^	0.0^c^	0.0^c^	3.8^c^		
12 h	475.4	328.5	1,666.7	315.5	438.2	192.3		
Butyrate								
3 h	0.0	3.3	0.0	0.0	13.9	0.0	29.42	0.0013
6 h	0.0	13.5	11.3	0.0	0.0	0.0		
9 h	20.3^a^	421.0^a^	248.6^a^	99.0^a^	181.4^a^	15.0^b^		
12 h	269.6	487.5	421.0	545.9	573.7	714.8		
*Branched-chain fatty acid*								
Isobutyrate								
3 h	0.0	3.4	1.6	0.0	2.7	1.7	1.54	<0.0001
6 h	0.0	7.3	3.9	2.2	2.2	3.0		
9 h	3.7^c^	24.9^a^	18.6^ab^	7.9^c^	11.6^bc^	12.1^b^		
12 h	2.1^d^	48.9^a^	45.6^a^	27.6^b^	18.2^c^	26.1^bc^		
Isovalerate								
3 h	0.0^b^	6.7^a^	2.7^ab^	0.4^b^	3.9^a^	2.6^ab^	5.90	< 0.0001
6 h	0.0^c^	15.7^a^	13.6^a^	5.4^ab^	4.0^bc^	4.5^bc^		
9 h	1.6^c^	50.5^a^	35.5^ab^	10.6^b^	13.5^ab^	15.5^ab^		
12 h	0.0^c^	159.2^a^	135.6^a^	75.9^ab^	34.4^b^	41.5^ab^		
Valerate								
3 h	0.1	0.0	0.0	0.0	0.2	0.0	0.37	0.0027
6 h	0.0	0.0	0.0	1.2	0.7	0.2		
9 h	0.8	0.8	0.0	0.3	0.6	0.1		
12 h	3.6^a^	1.3^b^	0.2^b^	1.0^b^	2.0^ab^	2.1^ab^		

^1^umol/g, expressed on a dry matter basis.

^2^Pooled SEM values

^a–d^Means in the same row with different superscript letters are different (*P* < 0.05).

#### Short-chain fatty acid production.

Significant treatment by time interactions were observed for acetate (*P* < 0.0001), propionate (*P* < 0.0001), and butyrate (*P* = 0.0013) productions (Table 4). No differences were observed for any SCFA after 3 or 6 h of fermentation. After 9 h of fermentation, acetate production values of BP and WYP (1,655.6 and 1,764.6 umol/g, respectively) were greater (*P* = 0.0053) than BBG and NBP (921.3 and 961.0 umol/g, respectively). After 12 h of fermentation, acetate production values for GB (1,571.7 umol/g) were the lowest (*P* = 0.0172) of all test substrates. Propionate production values for BP and WYP (157.7 and 126.1, respectively) were greater (*P* = 0.0147) than BBG, NBP, and GB (0.0, 0.0, and 3.8 umol/g, respectively) after 9 h, with no differences observed after 12 h of fermentation. Butyrate production values of all substrates were greater (*P* = 0.0253) than GB after 9 h, with no differences observed after 12 h or fermentation. The average molar ratios of acetate, propionate, and butyrate production values of all substrates after 9 and 12 h of fermentation were approximately 87:3:10 and 70:15:15, respectively.

#### Branched-chain fatty acid production.

Significant treatment by time interactions were observed for isobutyrate (*P* < 0.0001), isovalerate (*P* < 0.0001), and valerate (*P* = 0.0027) productions (Table 4). Isobutyrate production values for all substrates did not differ after 3 and 6 h of fermentation. After 9 h, isobutyrate values for WYP, GL, and GB (24.9, 18.6, and 12.1 umol/g, respectively) were greater (*P* = 0.0485) than BP (3.7 umol/g), with all test substrates having greater (*P* = 0.0001) values than BP after 12 h. Isovalerate production values for WYP were among the greatest in all time points (6.7, 15.7, 50.5, and 159.2 umol/g, respectively) with BP values among the lowest (0.0, 0.0, 1.6, and 0.0 umol/g, respectively). After 12 h, valerate production values for BP (3.6 umol/g) were greater (*P* = 0.0138) than WYP, GL, and BBG (1.3, 0.2, and 1.0 umol/g, respectively) with no differences observed among substrates for all other time points.

#### Gas production and composition.

Significant treatment by time interactions were observed for total gas (*P* = 0.0156), hydrogen (*P* < 0.0001), and methane (*P* = 0.0013) productions (Table 4). Total gas production values of BP, BBG, NBP, and GB (26.7, 17.2, 23.7, and 14.1 mL/g, respectively) were greater (*P* = 0.0427) than WYP (–0.9 mL/g) after 3 h of fermentation with no differences observed after any subsequent time points (Table 5). Hydrogen and methane production values were not different after 3 and 6 h of fermentation, with BP having greater (*P* < 0.0001) hydrogen production values after 9 and 12 h (720,323.0 and 391,566.1 nmol/g, respectively) and greater (*P* < 0.0001) methane production values after 12 h (3,384.2 nmol/g) than all test substrates.

**Table 5.** In vitro gas production of select legumes after 3, 6, 9, and 12 h using canine fecal inoculum

	Treatment							*P*
	BP	WYP	GL	BBG	NBP	GB	SEM	Trt × time
Total Gas, mL/g								
3 h	26.7^a^	–0.9^b^	9.1^ab^	17.2^a^	23.7^a^	14.1^a^	4.36	0.0156
6 h	22.6	11.6	11.6	14.9	14.2	20.8		
9 h	26.2	18.8	13.6	20.3	20.5	12.7		
12 h	32.7	29.4	15.9	20.8	28.0	15.3		
Hydrogen, nmol/g								
3 h	9,612.7	3,803.2	18,841.4	88,056.9	83,638.9	81,014.8	31,366.63	<0.0001
6 h	106,439.9	12,544.4	28,132.6	83,558.6	50,655.8	67,587.4		
9 h	720,323.0^a^	51,734.3^b^	11,877.7^b^	38,601.5^b^	65,683.5^b^	71,009.2^b^		
12 h	391,566.1^a^	2,898.7^b^	3,225.0^b^	18,384.0^b^	39,837.7^b^	50,386.0^b^		
Methane, nmol/g								
3 h	123.1	–127.6	163.9	319.5	223.9	569.3	242.24	<0.0001
6 h	785.5	–156.1	208.6	92.6	131.6	195.2		
9 h	6.6	325.9	817.3	338.5	644.7	–123.0		
12 h	3,384.2^a^	–85.0^b^	74.2^b^	142.2^b^	416.6^b^	229.0^b^		

^a–d^Means in the same row with different superscript letters are different (*P* < 0.05).

## DISCUSSION

### Ingredient Composition

Moisture, CP, and ash concentrations of WYP were in agreeance with those of Osei-Yaw and Powers (1986; 7.72%, 27.4%, 3.05%, respectively), Li and Ganjyal (2017; range: 9.2 to 10.2, 2.0 to 2.5, and 22.2 to 25.7, respectively), and Zhao et al. (2005; average: 9.1%, 22.4%, and 3.0% DMB, respectively). Similarly, concentrations of moisture, ash, and CP of GL were within range observed by Li and Ganjyal (2017; 8.0 to 10.0, 2.0 to 3.0, and 25.8 to 28.6, respectively). CP, AHF, and ash of BBG concentrations were slightly varied from those reported by Silva-Cristobal et al. (2008; 18.5, 1.6, and 5.4, respectively), however, IDF and SDF concentrations were similar (25.6 and 5.7, respectively). Concentrations of CP and starch of NBP were slightly less than those reported by Bourre et al. (2019; ranges: 25.3% to 26.1% and 38.2% to 40.1%, respectively). The USDA (2019) FoodData Central reports similar values for CP and ash of NBP (25.4% and 3.8%, respectively) and similar values or ranges for moisture, CP, fat, and ash of GB (7.7%, 2.5% to 25.8%, 6.5%, and 1.82% to 3.13%, respectively). Concentrations of CP, ash, AHF, SDF, and IDF of GB also were similar to those reported by Silva-Cristobal et al. (2008; 23.6%, 4.1%, 5.2%, 2.5%, and 18.3%, respectively).

Cereal beta-glucan concentrations were below the detection limit of the present assay (>0.35%). Total starch values of GL and GB were similar to those reported by Silva-Cristobal et al. (2008; 59.8% and 52.2%, respectively). The total starch value of WYP was similar to those reported by Eyaru et al. (2009; 57%). Lower resistant starch values were observed for GL, BBG, and GB then were reported by Silva-Cristobal et al. (2008; 7.4%, 11.4%, and 7.8%, respectively) and the value for WYP was considerably less than what was reported by Eyaru et al. (2009; 36.2%), most likely due to differences in methods of analysis. Herein, the resistant starch values were derived from direct analysis with 2N KOH following a 16 h digestion of non-resistant starch with an α-amylase/amylo-glucosidase mixture, whereas values reported by Silva-Cristobal et al. (2008) were derived following two sequential digestions with an α-amylase and amylo-glucosidase of 16 h and 45 m, respectively, and values reported by Eyaru et al. (2009) were derived indirectly by calculation of non-hydrolyzed/digested starch following only 120 m incubation with an amylase/amylo-glucosidase mixture.

It has been widely reported that legumes contain high proportions of sucrose and related oligosaccharides such as raffinose, stachyose, and verbascose (Sosulski et al., 1982; Saini and Gladstones, 1986; Saini, 1989; Apata, 2001; Njoumi et al., 2019). However, actual concentrations of these oligosaccharides may vary depending on multiple factors including growing conditions/stress (Jones et al., 1999), seed maturation (Saini, 1989), and cultivar (Martinez-Villaluenga et al., 2005). As a result of the monosaccharide components of sucrose and its related oligosaccharides, it was expected that both glucose and galactose would be of the highest concentrations in these legume seeds.

Some differences were expected between observed compositions and what has been reported in the literature. These are most likely a result of variations in growing and processing conditions, cultivar variety, and analysis methods, as mentioned previously.

### Metabolite Production via Third-Stage In Vitro Fermentation

The presence of a net negative change in pH and increases in volatile fatty acid and gas production over time are indicative of microbial activity (Cummings and Macfarlane, 1991) and thus, implicate a successful inoculation of test substrates.

The largest incremental changes from previous time points occurred after 9 and 12 h of fermentation for all analyzed fermentative end products. These data suggest that legumes are slowly fermentable substrates and thus, will be fermented toward the distal colon, and thus, may potentially provide greater amounts of carbohydrates to the distal colon, where proteolytic fermentation typically dominates (Macfarlane et al., 1992; Korpela, 2018). The largest decreases in pH were observed for both BBG and NBP after 12 h of fermentation.

The average molar ratios of acetate, propionate, and butyrate production values after 9 and 12 h of fermentation were within range, or slightly above those reported by Bergman (1990) for rumen and gastrointestinal tract microbial fermentation (range: 75:15:10 to 40:40:20). Values for GB were among the lowest producers of SCFA for all timepoints. Mallillin et al. (2008) analyzed the fermentative characteristics of a variety of legumes using human fecal inoculum over a period of 24 h and also noted that chick peas were among the lowest (*P* < 0.05) producers of acetate, propionate, and butyrate (2.8, 0.6, and 0.9 mmol/g fiber isolate, respectively) within the legumes category. Bednar et al. (2001) also analyzed a variety of legumes and observed greater (*P* < 0.05) total SCFA concentrations for lentils and split peas (8.5 and 8.7 mmol/g OM) than black and navy beans (7.2 and 6.8 mmol/g OM, respectively) after 7.5 h of fermentation using a canine ileal fluid inoculum. Our data illustrate a significant difference (*P* < 0.05) among acetate production values of similar substrates (WYP > BBG and NBP; *P* < 0.05) only after 9 h with numerically greater production values of acetate, propionate, and butyrate for WYP and GL after most other observed time points.

Overall, BCFA production values for these select legumes were relatively low, little valerate production during a 12 h in vitro fermentation period. The largest incremental increase among BCFA was observed for isovalerate production values after 12 h of fermentation. The BCFA values for GL and NBP were typically among the highest and lowest, respectively, of all test substrates. These data coincide with GL and NBP each having the greatest and lowest CP contents among test substrates, respectively. However, it is important to note that protein fermentation in vitro is hindered by the high availability of carbohydrates and reduced pH (Smith and Macfarlane, 1996), which may be an explanation for the low BCFA production values found.

Total gas production increased over 12 h of fermentation for all test substrates; however, hydrogen production values appeared to experience an increase and subsequent decrease after 9 or 12 h. Tubes from set 2 did not allow for the escape of microbially produced gasses, and the build-up of hydrogen may be detrimental to anaerobic microbial-fermentation via inhibition of NAD+ regeneration from NADH needed to continue non-methanogenic fermentation (McAllister and Newbold, 2008; Janssen, 2010). The observed hydrogen values alongside the continued increases in both SCFA and BCFA, suggest the reductions in hydrogen values were most likely a result of methanogenesis, whereby methanogens convert hydrogen into methane, which was markedly increased during the latter collection time points.

There are some limitations to the present study, including the determination of end products by difference of sugar blanks, whereby a microbiome rich in highly bioavailable sugars may result in an overestimated blank value, thus, resulting in lower calculated SCFA production values. The authors also acknowledge the inability to remove digested proteins prior to stage 3 fermentation may allow for an increase in proteolytic fermentation, however, as this is an opportunistic pathway for microbes, this would not become a major factor until later time points when available carbohydrates are depleted.

## CONCLUSIONS

These data suggest that legumes offer a diverse macronutrient with a fermentative profile similar to **BP**, a common fermentable fiber source used in the pet food industry. Additionally, legumes appear to be a slowly fermented, with the largest increases in fermentative activity occurring after 9 and 12 h of in vitro fermentation, which may have beneficial implications on the ratios of saccharolytic to proteolytic fermentation toward the distal colon. Future research should investigate the effects of legume-based diets on fermentative characteristics and microbial population in vivo and when added to a diet matrix.
